# TrackRefine: A Plug-and-Play Decoupled Enhancement Framework for Online Multi-Object Tracking and Segmentation

**DOI:** 10.3390/s26123696

**Published:** 2026-06-10

**Authors:** Longfei Qie, Chunlei Chai, Ruixue Wang, Chao Bi, Ruiqi Ma, Aijun Zhang, Jiakui Tang

**Affiliations:** 1 School of Mechanical and Electrical Engineering, Beijing University of Chemical Technology, Beijing 100029, China; qlf@buct.edu.cn (L.Q.); 2024210423@buct.edu.cn (C.C.); wrx@buct.edu.cn (R.W.); bichao@mail.buct.edu.cn (C.B.); 2025210430@buct.edu.cn (R.M.); 2 College of Resources and Environment, University of Chinese Academy of Sciences, Beijing 100049, China

**Keywords:** multi-object tracking and segmentation, mask refinement, multimodal memory bank, trajectory association, YOLO, plug-and-play framework

## Abstract

**Highlights:**

**What are the main findings?**

- We present TrackRefine, a plug-and-play, decoupled framework that enhances online multi-object tracking and segmentation without requiring modifications to the front-end instance segmenter or additional end-to-end joint training.
- Improving mask quality, memory reliability, and progressive association jointly is beneficial for enhancing robustness in complex scenes with occlusion, dense targets, and long-term trajectory interruptions.

**What are the implications of the main findings?**

- The experimental results demonstrate that online MOTS performance can be effectively improved through modular back-end enhancement rather than relying solely on end-to-end joint training.
- Owing to its plug-and-play and decoupled characteristics, TrackRefine is easier to deploy, migrate, and extend in real-world applications such as autonomous driving, surveillance, and robotic perception.

**Abstract:**

Multi-object tracking and segmentation (MOTS) aims to jointly perform pixel-level instance segmentation and temporal identity association for multiple objects in video sequences. Existing online decoupled MOTS methods face several challenges in complex scenarios, including limited front-end mask quality, corruption of memory representations under prolonged occlusion, and unstable data association and trajectory recovery. To address these limitations, we propose TrackRefine, a plug-and-play decoupled enhancement framework. TrackRefine enhances overall performance through back-end refinement without modifying the architecture of the front-end instance segmenter or relying on additional end-to-end joint training. Specifically, we introduce a lightweight Fast GrabCut-based mask refinement module to optimize mask boundaries, a multimodal long-short-term memory bank that integrates appearance, semantic, and shape cues for identity modeling, and a progressive three-stage association strategy for stable matching and long-term trajectory recovery. Experimental results on MOTS20 show that TrackRefine achieves 69.4 sMOTSA, 82.7 MOTSA, and 478 Frag. Experimental results on KITTI MOTS show that it achieves 62.4/73.7 sMOTSA and 78.0/85.4 MOTSA for pedestrians and cars, respectively. Extensive experiments with different front-end instance segmenters verify its plug-and-play flexibility and decoupled design, while ablation studies confirm the effectiveness of each core module. These results show that TrackRefine provides an efficient and practical solution for online MOTS in complex scenarios.

## 1. Introduction

Multi-object tracking and segmentation (MOTS) aims to jointly perform pixel-level instance segmentation of multiple objects in videos and associate their identities across time, representing an important extension of multi-object tracking from bounding-box-level to pixel-level representations [1]. Compared with conventional multi-object tracking (MOT), which describes object locations only with bounding boxes, MOTS provides richer information on object contours, occlusion relationships, and spatiotemporal structures [2,3,4]. By generating pixel-level object masks along with temporally consistent identities, MOTS allows sensors—such as cameras and radar—to extract more precise and fine-grained information from dynamic scenes. This capability not only improves the interpretation of scene geometry and motion but also directly benefits downstream modules in autonomous systems, intelligent surveillance, and robotic perception.

Since Voigtlaender et al. [5] introduced the MOTS task and established benchmarks such as KITTI MOTS and MOTSChallenge, this research direction has attracted sustained attention. Unlike conventional MOT, which primarily focuses on detection and association, MOTS explicitly incorporates mask quality into the evaluation process. Therefore, segmentation is not merely an auxiliary output, but a key component that directly determines the upper bound of system performance. For online MOTS, achieving a balance among inference efficiency, mask quality, identity consistency, and trajectory recovery capability remains a challenging problem [6].

Existing MOTS methods can generally be divided into two categories: one category consists of joint modeling approaches, exemplified by TrackR-CNN, which use a unified framework to simultaneously perform detection, segmentation, and association [5]; the other is the decoupled tracking-by-segmentation approach, which separates the front-end instance segmentation from the back-end temporal association to achieve greater modular flexibility and engineering scalability [7,8,9]. Compared to the joint modeling approach, decoupled online MOTS has two main advantages: it can directly benefit from advances in front-end instance segmenters and allows the back-end association module to be optimized independently. As a result, segmentation-driven and decoupled tracking frameworks have become an important research direction in recent years [10]. However, in complex real-world scenarios, existing decoupled online methods still face three main challenges.

First, front-end mask quality remains a major bottleneck that limits the upper bound of system performance. Existing decoupled methods typically feed the raw outputs of the instance segmenter directly into the subsequent tracking module. While this maintains the independence of the modules, it also makes the overall performance highly dependent on the quality of the front-end masks. In scenarios involving fast motion, blurred boundaries, touching instances, and partial occlusion, real-time instance segmenters tend to produce coarse boundaries, local missing regions, or insufficient instance separation. These inaccuracies not only reduce mask IoU and affect key metrics such as sMOTSA, but also contaminate the extracted features, thereby weakening cross-frame association stability. Therefore, how to effectively enhance the initial mask quality without compromising online processing and decoupling characteristics is an important issue in online MOTS.

Second, memory modeling under long-term occlusion remains insufficiently robust. To maintain identity consistency when an object reappears after temporary disappearance or occlusion, online tracking and video object segmentation methods usually introduce explicit memory mechanisms to store historical information [11,12]. However, most existing methods rely on a single appearance feature or simple geometric constraints for modeling. When objects are visually similar, undergo non-rigid deformation, or are affected by illumination changes and motion blur, a single feature often fails to provide stable discriminative cues [12]. Furthermore, when objects are severely occluded or only partially visible, the currently observed features often contain considerable background and occlusion noise. If such low-quality features are continuously written into the memory bank, historical representations may gradually drift, thereby degrading subsequent re-identification and trajectory recovery. Therefore, online MOTS requires an enhanced memory mechanism that jointly supports multi-source information fusion and selective updating.

Third, data association and lost trajectory recovery under complex conditions remain prone to failure. Existing online MOTS methods typically adopt the two-stage association strategy widely used in online MOT [6]. The first stage performs strongly constrained matching based on motion prediction, IoU, and high-confidence detections, while the second stage performs supplementary association using low-confidence detections or relaxed appearance similarity. This strategy is generally effective in conventional scenarios; however, under conditions such as rapid non-rigid deformation, severe camera motion, or target reappearance after long-term occlusion, the geometric continuity between adjacent frames significantly deteriorates, preventing successful matching in the first stage. If subsequent matching relies solely on relaxed appearance similarity, it is prone to erroneous associations, resulting in identity switches and trajectory fragmentation. Although prior works have begun exploring finer-grained cascade association strategies [13], these methods are mainly designed for box-level MOT or general instance association. Their intermediate association stages often remain limited to threshold relaxation or detection-confidence partitioning and do not explicitly function as candidate filtering and transitional recovery stages for online MOTS.

To address the aforementioned challenges, this paper proposes TrackRefine, a plug-and-play decoupled enhancement framework for online MOTS. TrackRefine is designed to be seamlessly integrated into existing tracking-by-segmentation pipelines without modifying the front-end instance segmenter or requiring additional end-to-end joint training. It improves overall system performance through three components: lightweight mask refinement, enhanced multimodal memory modeling, and a progressive three-stage association strategy. Compared with existing methods, our approach emphasizes the compatibility and interchangeability of the back-end enhancement modules with different front-end instance segmenters, making TrackRefine not only competitive in performance but also more suitable for practical online deployment and system migration. The main contributions of this paper are summarized as follows:

(1) A lightweight mask refinement module based on Fast GrabCut is introduced, locally refining initial masks without altering the front-end instance segmenter, thereby providing more stable foreground representations for subsequent feature extraction and cross-frame association.
(2) An enhanced multimodal long-short-term memory bank is constructed to jointly model appearance, semantic, and shape information. By integrating selective updating with a quality-aware gating mechanism, memory contamination caused by occlusion and background interference is alleviated, thereby improving identity discrimination when targets reappear after long-term occlusion.
(3) A progressive three-stage association strategy is designed, in which a dynamic buffering and candidate filtering mechanism is introduced in the second stage, and additional spatial constraints are imposed during long-term recovery, enabling stable progressive association from high-confidence geometric matching to long-term lost trajectory recovery.

## 2. Background and Motivation

### 2.1. Multi-Object Tracking and Segmentation Methods

Early MOTS studies predominantly employed joint modeling frameworks. Representative methods, such as TrackR-CNN [5] and recent end-to-end MOT frameworks [14], extend detection and instance segmentation networks with temporal modeling and association branches, attempting to jointly perform detection, segmentation, and tracking within a unified network. More recently, end-to-end transformer-based MOT frameworks, such as CO-MOT [15], have further explored unified detection-association optimization through transformer architectures and label-assignment strategies. These approaches can share feature representations within a single framework; however, the tight coupling among detection, segmentation, and association modules renders training and optimization relatively complex and limits the flexibility to independently incorporate advances in the front-end segmenter or back-end associator.

In contrast, decoupled methods separate front-end instance segmentation from back-end temporal association, thereby achieving greater modular flexibility and engineering scalability, and have gradually become an important research direction in MOTS in recent years. Representative works include MPNTrackSeg [16], PointTrack [17], and OPITrack [18]. These methods typically first generate frame-wise instance masks using an instance segmenter and then perform cross-frame identity association by combining appearance cues, geometric cues, graph-optimization strategies, or graph representation learning. Among them, MPNTrackSeg leverages a message-passing network for global graph optimization, PointTrack improves online association efficiency through point-set representations, and OPITrack further exploits the discriminative role of point-set structures in MOTS. UniTrack [19] explores graph-based representation learning for multi-object tracking and can be regarded as a related effort in association modeling. Overall, decoupled frameworks offer clear advantages in deployment convenience, replaceability, and engineering transferability, but their performance also depends critically on the combined effectiveness of front-end mask quality and back-end association design.

### 2.2. Mask Quality and Mask Refinement in Online MOTS

High-quality masks can provide cleaner foreground regions for appearance modeling, shape modeling, and data association under occlusion, thereby improving overall tracking stability. Most existing decoupled methods directly use the raw outputs of instance segmentation networks as the final masks [9]. However, in scenarios involving fast motion, blurred boundaries, adjacent instance contact, or severe occlusion, these masks are prone to rough edges, local missing regions, or adhesion between adjacent instances, which in turn degrades mask IoU and weakens the robustness of subsequent association.

In recent years, some studies have attempted to improve mask quality with the aid of foundation segmenters, such as SAM [20], its video version, SAM 2 [21], and SAM-based matching frameworks [1], which have shown strong capabilities in zero-shot and promptable segmentation. However, such methods are typically characterized by large model sizes and high inference costs, making them difficult to deploy directly in online MOTS scenarios with lightweight requirements. On the other hand, recent approaches introduce video-level context through spatiotemporal memory propagation, segmentation-driven tracking, or unified tracking-and-segmentation modeling to improve the temporal consistency and boundary quality of segmentation results [9,10]. Although these methods can exploit cross-frame information more effectively, they usually rely on more complex inter-frame feature interactions, memory maintenance, or optimization procedures, and therefore struggle to simultaneously satisfy the requirements of speed, stability, and plug-and-play characteristics in online settings.

By contrast, lightweight post-processing methods that require no additional training and can be directly applied to local regions are more suitable as mask enhancement modules in decoupled online frameworks. Among such methods, GrabCut and fully connected conditional random fields (CRFs) are two representative strategies. CRFs can enhance local smoothness and low-level edge consistency, but their adaptability remains relatively limited in complex backgrounds and fine-grained boundary scenarios [22]. GrabCut, in contrast, jointly models foreground and background distributions through graph-cut optimization, offering stronger capability in foreground–background separation and boundary fitting [23]. However, systematic exploration of such traditional refinement methods in online MOTS remains limited, particularly with respect to lightweight adaptation for decoupled frameworks. Motivated by this, this paper proposes a lightweight mask refinement strategy based on Fast GrabCut, which performs graph-cut optimization only within the local region of interest (RoI) of each target and controls computational cost through scale constraints, automatic trimap initialization, and single-iteration refinement.

### 2.3. Memory Mechanisms and Multimodal Feature Representations

Previous works, such as MeMOT [24] and MeMOTR [11], have demonstrated that explicitly modeling long-term memory helps mitigate association interruptions caused by occlusion, prolongs the temporal span of reliable target matching, and improves the consistency and stability of identity representations. Nonetheless, most existing online tracking methods still primarily rely on a single appearance feature or simple geometric priors for target representation [25], while recent semantic-aware tracking studies indicate that semantic information can provide complementary cues for identity association in complex scenes [26]. While such designs are efficient in conventional scenarios, they often fail to provide stable discriminative cues in situations with highly similar appearances, drastic pose changes, partial visibility, or significant motion blur. The introduction of DanceTrack [27] emphasizes the challenges posed by scenarios with “similar appearance but complex motion” for traditional association paradigms, and Focus on Details [28] further highlights that finer-grained and more diverse representations are crucial for online tracking in complex scenarios.

Beyond limitations in representation capacity, existing memory-enhanced methods also face potential noise accumulation during memory updates. Some approaches adopt recursive feature updates to maintain historical representations, which helps incorporate longer-term temporal information but also allows the quality of current observations to continuously influence memory content [11]. When a target is severely occluded, partially visible, or suffers from degraded detection quality, the extracted features are often contaminated with background and occlusion noise; without explicit quality-aware write mechanisms, historical representations may gradually drift, undermining matching reliability upon target reappearance and increasing the risk of identity switches.

### 2.4. Data Association and Lost Trajectory Recovery

Data association is a core component of online MOTS, aiming to stably match current detections with historical trajectories across consecutive video frames [29]. Traditional online trackers commonly employ a two-stage association strategy based on motion prediction and appearance features. Representative methods such as DeepSORT [25] first combine Kalman filtering with deep appearance metrics for matching; ByteTrack [30] further demonstrates the value of low-confidence detections in maintaining trajectory continuity, significantly improving online tracking performance through cascade matching; BoT-SORT [31] additionally incorporates mechanisms such as camera motion compensation to enhance positional alignment in complex scenarios.

Although two-stage association is generally effective in conventional scenarios, it remains prone to failure under fast non-rigid target deformations, severe camera motion, or reappearance after prolonged occlusion. On one hand, the IoU between adjacent frames may drop significantly, preventing correct geometric or motion-constrained matching in the first stage. On the other hand, when a target reappears, relying solely on relaxed appearance matching can lead to erroneous associations due to feature drift or similar appearances. Recent works have begun exploring three-stage or finer-grained cascade matching strategies [32,33], which improve association performance by hierarchically handling active trajectories, temporarily lost trajectories, and long-term inactive trajectories. While promising, the intermediate stages of existing methods are often limited to simple threshold relaxation or detection confidence stratification and do not effectively perform candidate pruning, transitional filtering, or preparatory recovery. As a result, the subsequent recovery stage may still receive a substantial number of noisy candidates, increasing the risk of incorrect trajectory stitching. To address this limitation, this paper proposes a progressive three-stage association strategy, designing the second stage as a dynamic buffer that not only provides supplementary matching but also dynamically adjusts candidate filtering according to trajectory mismatch status, thereby establishing a higher-purity candidate pool for the third-stage long-term recovery.

## 3. Methods

### 3.1. Overall Framework

**Figure 1:**
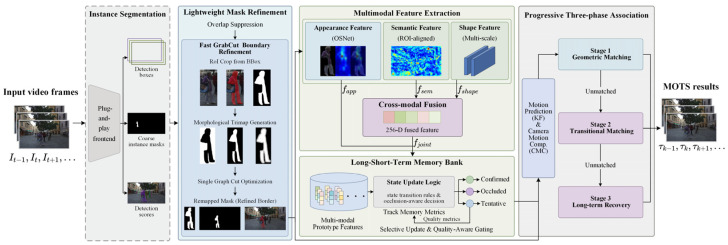
Overall framework of TrackRefine for online MOTS. Arrows indicate the data flow between modules, and different background colors indicate different functional modules of the framework, including instance segmentation, mask refinement, multimodal feature extraction, memory bank updating, and progressive three-phase association.

Given an input video sequence V, where V denotes the set of all video frames in the input sequence, it can be represented as

(1) $$V=\{I_{t}{\}}_{t=1}^{T},$$

where $I_{t}$ denotes the t-th video frame and T is the total number of frames. The system first employs an instance segmenter to generate a set of detections for each frame. The detection set of frame t is denoted as

(2) $$D_{t}=\{(b_{i}^{t},m_{i}^{t},c_{i}^{t},s_{i}^{t}){\}}_{i=1}^{N_{t}},$$

where $D_{t}$ denotes the set of all detected instances in frame t, and $N_{t}$ is the total number of detected instances in that frame. For the i-th detected instance in frame t, $b_{i}^{t}$, $m_{i}^{t}$, $c_{i}^{t}$, and $s_{i}^{t}$ denote its bounding box, binary segmentation mask, category label, and detection confidence score, respectively. Subsequently, the system sequentially performs mask refinement, multimodal feature encoding, long-short-term memory updating, and progressive three-stage association. Finally, it outputs a set of target trajectories

(3) $$T=\{\tau_{k}{\}}_{k=1}^{K},$$

where T denotes the set of all tracked trajectories, K denotes the total number of generated trajectories and $\tau_{k}$ represents the k-th trajectory with pixel-level masks and temporally consistent identity labels. The overall framework of TrackRefine for online MOTS is illustrated in Figure 1.

The entire framework consists of three core components:

1. a lightweight mask refinement module for improving the quality of initial instance masks;
2. an enhanced multimodal long-short-term memory bank for maintaining more stable identity representations during long-term occlusion and target reappearance; and
3. a progressive three-stage association strategy for achieving hierarchical association from high-confidence geometric matching to long-term lost-track recovery.

All three components are built upon the generic detection boxes, instance masks, and their derived features produced by the front-end, without relying on any specific front-end network architecture.

Algorithm 1 summarizes the complete pipeline of TrackRefine. For each frame, the front-end instance segmenter first produces frame-wise detections, including bounding boxes, masks, class labels, and confidence scores. The proposed framework then suppresses duplicate masks and refines the remaining initial masks using a lightweight local Fast GrabCut module. Based on the refined masks, multimodal features are extracted from appearance, semantic, and shape cues. Existing trajectories are predicted with Kalman filtering and camera motion compensation, and are subsequently matched with current detections through the proposed progressive three-stage association strategy. After matching, trajectory states are updated, and the long-short-term memory bank is selectively updated according to observation quality. This design integrates mask refinement, multimodal memory modeling, and progressive association into a unified online MOTS pipeline while preserving the plug-and-play and decoupled nature of TrackRefine. Detailed pseudocode descriptions of the individual modules are further provided in Algorithms S1–S5 in the Supplementary Materials.
**Algorithm 1.**  Overall Pipeline of TrackRefine**Input:** Video sequence $V=\{I_{t}{\}}_{t=1}^{T}$, front-end instance segmenter Φ, memory bank M, thresholds Θ **Output:** Trajectory set T 1  Initialize T←∅ and M←∅ 2  **for** t =1 **to** T **do** 3     Obtain frame-wise detections:$D_{t}=\Phi (I_{t})=\{(b_{i}^{t},m_{i}^{t},c_{i}^{t},s_{i}^{t}){\}}_{i=1}^{N_{t}}$4     Suppress highly overlapped duplicate masks in $D_{t}$ 5     Refine initial masks by local Fast GrabCut:D^t=MaskRefine(It,Dt)6     Extract appearance feature $f_{i}^{app,t}$, semantic feature $f_{i}^{sem,t}$, and shape feature $f_{i}^{shp,t}$ from D^t 7     Fuse semantic and shape cues:$f_{i}^{joi,t}=JointFeatureEncode\left(f_{i}^{sem,t},f_{i}^{shp,t}\right)$8     Construct multimodal feature:$F_{i}^{t}=\left(f_{i}^{app,t},f_{i}^{joi,t}\right)$9     Construct the multimodal feature set:$F_{t}=\{F_{i}^{t}{\}}_{i=1}^{N_{t}}$10     Predict existing trajectory states using Kalman filtering and camera motion compensation 11   Partition trajectories into active, recently unmatched, and lost states 12       Perform progressive three-stage association:At,Ut=ThreeStageAssociate(T,D^t,Ft,M)13   **for each** matched pair $(\tau_{k},d_{j})\in A_{t}$ **do** 14           Update the state, box, mask, and identity of $\tau_{k}$ using $d_{j}$ 15           Estimate the observation quality $Q_{j}^{t}$ 16             Selectively update short-term memory and long-term prototypes:$M_{k}\leftarrow QualityAwareUpdate(M_{k},d_{j},F_{j}^{t},Q_{j}^{t})$17    **end for** 18    Mark unmatched trajectories as occluded or lost according to missing duration 19    Initialize new tentative trajectories for unmatched detections in $U_{t}$ 20    Remove expired trajectories from T 21 **end for** 22  return T


### 3.2. Lightweight Mask Refinement

#### 3.2.1. Instance Overlap Suppression

For any two detection masks within the same frame, we compute the mask IoU ${IoU}_{mask}$ as follows:

(4) $${IoU}_{mask}(m_{i}^{t},m_{j}^{t})=\frac{|m_{i}^{t}\cap m_{j}^{t}|}{|m_{i}^{t}\cup m_{j}^{t}|},$$

where $m_{i}^{t}$ and $m_{j}^{t}$ denote the binary masks corresponding to the i-th and j-th detected instances in frame t, respectively. The operators ∩ and ∪ denote the intersection and union of the mask pixel sets, respectively, and ∣⋅∣ represents the foreground pixel area of a mask. $\theta_{m}$ is the instance-overlap suppression threshold; a larger value of $\theta_{m}$ indicates a more conservative duplicate-removal strategy. If

(5) $${IoU}_{mask}(m_{i}^{t},m_{j}^{t})>\theta_{m},\theta_{m}\in (0,1)$$

it is considered that both masks correspond to duplicate instances of the same object. In this case, we retain the detection with the higher confidence and suppress the lower-confidence redundant instance to reduce fragmentation and false positives, minimizing interference with subsequent associations.

#### 3.2.2. Mask Refinement Based on Fast GrabCut

After instance overlap suppression, we further apply a local boundary refinement to the retained initial masks using the graph-cut optimization idea of GrabCut [8]. Since applying conventional GrabCut to the entire frame is computationally expensive, we adopt a lightweight variant based on a “local RoI + single graph-cut optimization” strategy.

Specifically, for each detection instance, the system first crops a RoI from the original image based on the instance’s bounding box, including the target and its local context. If the RoI size exceeds a preset limit $R_{max}$, it is resized proportionally to the target resolution to control the computational cost of the graph-cut optimization. Then, based on the initial binary mask, a trimap is automatically constructed using morphological erosion and dilation: the eroded area is considered the foreground, the dilated boundary region is the unknown area, and the rest is the background. Afterward, a single graph-cut optimization is performed only within this local region. Finally, the optimized mask is mapped back to the original image size, resulting in a sharper boundary and clearer instance separation.

**Figure 2:**
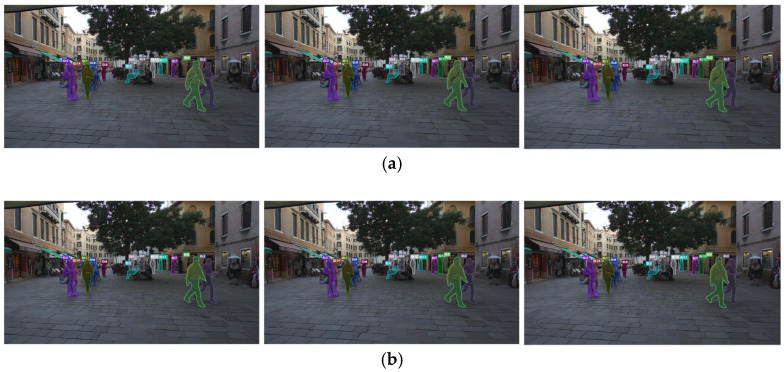
Visualization of mask refinement results. Different colors denote different tracking IDs, with the same color representing the same tracked object across consecutive frames. (**a**) Before refinement. (**b**) After refinement.

Compared to multi-iteration optimization schemes, Fast GrabCut retains the boundary fitting and foreground–background separation advantages of GrabCut while controlling its computational cost within a range suitable for online MOTS. Figure 2 shows the visualization of the mask refinement results before and after optimization. As can be seen, the introduced mask refinement strategy improves issues such as rough instance boundaries, local omissions, and insufficient separation between adjacent targets to some extent.

### 3.3. Enhanced Multimodal Long Short-Term Memory Bank

#### 3.3.1. Multimodal Feature Representation

For each detection instance, we construct a multimodal representation consisting of appearance, semantic, and shape features. For the appearance branch, a lightweight re-identification network, OSNet, is employed to extract appearance features of instances. Specifically, the refined instance mask is used to retain the foreground pixels at the instance level, preserving only the relevant regions of the target. The cropped instance image is then fed into OSNet to obtain an appearance feature vector $f_{i}^{app}\in R^{512}$. For the semantic branch, intermediate feature maps from the front-end detection backbone are used to extract local features corresponding to the target instance through RoI Align, followed by global average pooling and channel alignment to obtain a semantic feature $f_{i}^{sem}\in R^{512}$. For the shape branch, the binary mask is scaled to a fixed resolution and input into a lightweight multi-scale mask encoder to extract shape features $f_{i}^{sha}\in R^{256}$ that represent the target’s contour topology and local geometric structure.

Considering that semantic and shape information are strongly complementary in complex scenarios, we concatenate them as $z_{i}=[f_{i}^{sem};f_{i}^{sha}]\in R^{768}$, and use a linear projection layer followed by a Transformer encoder for cross-modal interaction, generating a compact joint feature representation $f_{i}^{joi}=\mathrm{Transformer}(\mathrm{Proj}(z_{i}))\in R^{256}$. Ultimately, the appearance feature $f_{i}^{app}$ and the joint semantic-shape feature $f_{i}^{joi}$ form the core representations for subsequent memory updates and cross-frame matching.

#### 3.3.2. Long-Short-Term Memory Structure

Based on multimodal representations, we maintain the memory state for the k-th trajectory $M_{k}$ as:

(6) $$M_{k}=\{S_{k},P_{k}\},$$

where $S_{k}$ denotes the short-term memory queue used to preserve local temporal context, and $P_{k}$ denotes the long-term prototype set used to describe the stable identity representation of the trajectory over a longer time span. Specifically, we define

(7) $$P_{k}=\{P_{k}^{app},P_{k}^{joi}\},$$

where $P_{k}^{app}$ denotes the long-term appearance prototype and $P_{k}^{joi}$ denotes the long-term joint semantic-shape prototype. The short-term memory emphasizes recent variations, while the long-term prototypes emphasize stable identity. Together, they support matching and recovery under complex occlusion and reappearance scenarios.

#### 3.3.3. Selective Update

For successfully matched trajectories, we perform smooth updates on the long-term prototypes as:

(8) $$P_{k}^{\left(t\right)}=\alpha P_{k}^{\left(t-1\right)}+(1-\alpha )f_{k}^{\left(t\right)},\left.\alpha \in [0,1\right)$$

where $P_{k}^{\left(t\right)}$ denotes the long-term prototype of trajectory k at frame t, $f_{k}^{\left(t\right)}$ denotes the target feature extracted from the current frame, and α is the momentum coefficient. The coefficient α determines the relative contribution of the historical prototype and the current observation during the update. Specifically, a larger α assigns a higher weight to the previous prototype $P_{k}^{\left(t-1\right)}$, making the updated prototype more dominated by long-term historical information and therefore more stable. In contrast, a smaller α increases the contribution of the current feature $f_{k}^{t}$, making the prototype more responsive to recent appearance variations. For the long-term prototype set $P_{k}$, this momentum-based update rule is applied to both the appearance prototype $P_{k}^{app}$ and the joint semantic-shape prototype $P_{k}^{joi}$.

In online scenarios, not all observations are suitable for writing into long-term memory. When a target is severely occluded, partially visible, or suffers from significant detection quality degradation, the features extracted from the current frame often contain more background and occlusion interference. If directly written into the long-term prototype, this could lead to identity drift. To address these practical reliability issues, we introduce two-level constraints.

First, we adopt a selective write strategy based on trajectory state. Long-term memory updates are performed only for confirmed trajectories; for occluded trajectories, only conservative low-frequency updates are performed; and for tentative trajectories, long-term memory updates are not performed. This strategy helps maintain memory freshness while reducing the contamination of long-term prototypes by unstable observations.

Second, a quality-aware gating mechanism is introduced. A feature is written into the long-term prototypes only when the corresponding instance exhibits sufficiently reliable mask area, detection quality, and motion consistency. Otherwise, the update is restricted to the short-term state to reduce noise accumulation caused by occlusion, background leakage, and abnormal speed disturbances.

#### 3.3.4. Overall Pipeline of Memory Bank Update Mechanism

**Figure 3:**
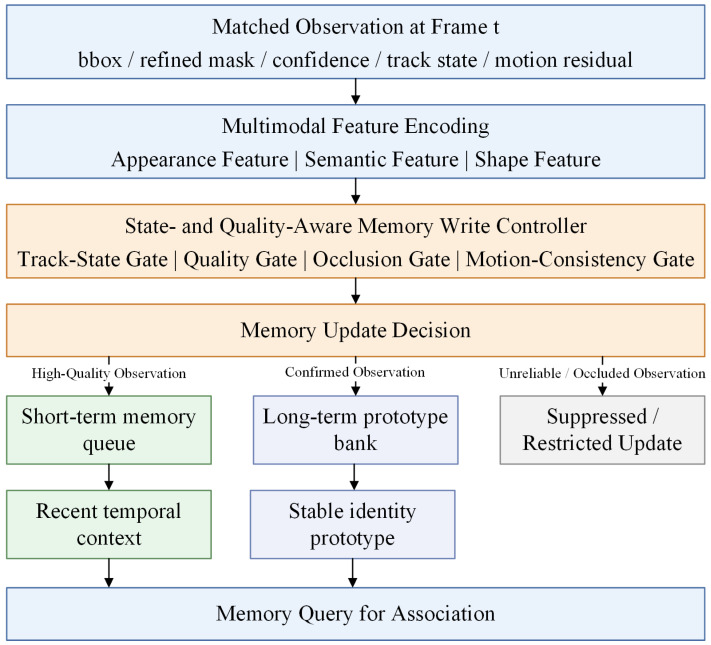
Quality-aware long-short-term memory update mechanism. Arrows indicate the decision flow of memory updating. Different colors distinguish different functional stages: feature encoding, gate-based memory write control, memory update decision, short-term memory update, long-term prototype update, and suppressed/restricted update.

To better illustrate the working process of the above modules, the memory bank update mechanism is shown in Figure 3. The update mechanism jointly considers trajectory state, observation quality, occlusion status, and motion consistency to determine whether the current observation should be written into short-term memory, long-term prototypes, or suppressed to prevent memory contamination.

The matched observation at frame t, including the bounding box, refined mask, confidence score, trajectory state, and motion residual, is first encoded into multimodal appearance, semantic, and shape representations. These features are then evaluated by a state- and quality-aware memory write controller composed of a track-state gate, quality gate, occlusion gate, and motion-consistency gate. According to the reliability of the current observation, the controller determines whether the observation should be written into the short-term memory queue, used to update the long-term prototype bank, or suppressed/restricted to avoid memory contamination. High-quality observations preserve recent temporal context, confirmed observations maintain stable long-term identity prototypes, and unreliable or occluded observations are prevented from directly corrupting the long-term memory. The updated memory representations are subsequently queried for data association.

In this way, unreliable observations from tentative trajectories, severe occlusion, or abnormal motion are prevented from directly contaminating the long-term prototypes. Reliable confirmed observations update both the appearance prototype and the joint semantic-shape prototype through a momentum rule, whereas low-quality observations are retained only in the short-term memory.

### 3.4. Progressive Three-Stage Association Strategy

**Figure 4:**
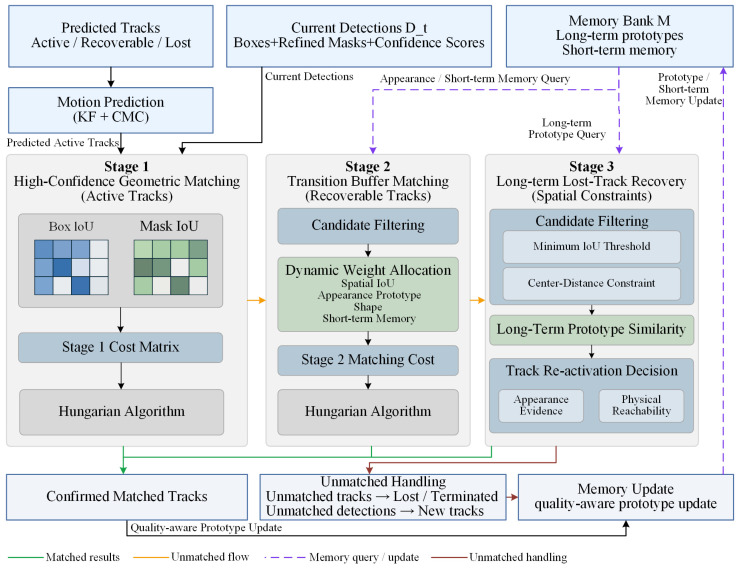
Illustration of the progressive three-stage association strategy.

For each active trajectory, we first apply Kalman filtering (KF) to predict the trajectory state and then use camera motion compensation (CMC) to apply global affine correction to the predicted position, thereby mitigating position offsets caused by camera shake and vehicle motion [6]. Next, according to the current trajectory state, the association process is divided into three stages to perform matching. As shown in Figure 4, the proposed three-stage association strategy consists of high-confidence geometric matching, transition buffering, and long-term recovery. Compared with two-stage matching, the proposed strategy explicitly separates short-term continuation, transitional buffering, and long-term reactivation.

#### Progressive Three-Stage Matching Strategy

The first stage focuses on active trajectories with strong spatial continuity and employs a high-confidence matching strategy dominated by geometric consistency. The system computes the IoU of predicted bounding boxes and mask IoU between the predicted trajectory and the current detection. Prior to cost matrix construction, the two similarity scores are normalized to a unified scale to reduce the impact of differences in score ranges on matching assignment. The cost matrix for the first stage is defined as:

(9) $$C_{ij}^{\left(1\right)}=1-\lambda_{1}{\tilde{\mathrm{IoU}}}_{box}-\lambda_{2}{\tilde{\mathrm{IoU}}}_{mask},$$

where $C_{ij}^{\left(1\right)}$ denotes the matching cost between the i-th trajectory and the j-th detection in the first stage, ${\tilde{IoU}}_{box}$ and ${\tilde{IoU}}_{mask}$ denote the normalized bounding-box IoU and mask IoU, respectively, and $\lambda_{1}$ and $\lambda_{2}$ are the corresponding weights. The Hungarian algorithm is used to achieve optimal assignment based on the cost matrix. To avoid the risk of mismatching due to amplified relative score differences after normalization, we apply an additional absolute spatial constraint: only assignments satisfying the original bounding-box IoU constraint ${\mathrm{IoU}}_{box}\geq 0.5$ are accepted. Candidates that do not pass this constraint are retained for processing in the subsequent stages.

For trajectories that were not successfully matched in the first stage but still have the potential for recovery, we introduce a second stage as the buffer stage that transitions from geometry-dominated matching to feature-dominated matching. Unlike existing methods, which only relax thresholds for supplementary matching, we explicitly design the second stage as an intermediate stage for “candidate filtering + matching transition.” The matching cost in the second stage is composed of spatial IoU, appearance prototype similarity, shape similarity, and short-term memory similarity:

(10) $$C_{ij}^{\left(2\right)}=w_{iou}C_{ij}^{iou}+w_{app}C_{ij}^{app}+w_{sha}C_{ij}^{sha}+w_{stm}C_{ij}^{stm},$$

where $C_{ij}^{\left(2\right)}$ denotes the matching cost between the i-th trajectory and the j-th detection in the second stage, $C_{ij}^{iou}$, $C_{ij}^{app}$, $C_{ij}^{sha}$, and $C_{ij}^{stm}$ denote the geometric cost, appearance cost, shape cost, and short-term memory cost between the i-th trajectory and the j-th detection, respectively. $w_{iou}$, $w_{app}$, $w_{sha}$, and $w_{stm}\in [0,1]$ are the corresponding weights, satisfying $w_{iou}+w_{app}+w_{sha}+w_{stm}=1.$

To adapt to different mismatch states, we apply a piecewise dynamic weight allocation strategy based on the number of mismatch frames Δt since the last successful match: when Δt∈[0,1], the trajectory still has strong spatial continuity, so geometric constraints dominate; when Δt∈(1,5], the importance of shape information is gradually increased; and when Δt∈(5,20], appearance and shape features become the main basis for matching in the second stage. This segmentation design aligns with the gradual evolution of mismatch states in online MOTS, from short-term local disturbances to long-term identity recovery. To avoid low-quality candidates entering the subsequent recovery process, the second stage retains only candidate matches that satisfy basic geometric or appearance feasibility constraints and passes the unmatched trajectories to the third stage.

The third stage is used to recover long-term unmatched trajectories. At this stage, relying solely on appearance similarity is risky because, after long-term occlusion, targets may undergo significant pose changes, partial occlusion, and background interference. Simple feature matching can lead to incorrect stitching. For this stage, we no longer rely purely on appearance similarity but apply strict spatial constraints before memory feature matching. Specifically, candidates are only allowed to participate in long-term prototype similarity evaluation and trajectory reactivation decision-making if they satisfy the minimum IoU threshold and center-point distance constraints. By combining appearance evidence with spatial feasibility, the third stage enables more robust identity reactivation after long-term occlusion and reduces the risk of identity switches during recovery.

Overall, the core idea behind the three-stage association is not simply to increase the number of matching rounds but to gradually adjust the matching basis according to trajectory state changes: the first stage emphasizes high-confidence geometric consistency, the second stage serves as a transition buffer and candidate filtering, and the third stage performs long-term recovery under spatial reachability constraints. This design allows the system to apply more appropriate association strategies under different mismatch conditions.

## 4. Experiments

### 4.1. Experimental Settings

TrackRefine was implemented based on the PyTorch framework (PyTorch 2.5.1, Meta AI, Menlo Park, CA, USA), and all experiments were conducted on a single NVIDIA RTX 4090 GPU. In the front-end detection and segmentation module, to verify the decoupling capability and replaceability of TrackRefine, Ultralytics YOLO-series instance segmenters [34] were adopted as the unified front-end, with publicly available pre-trained weights initialized on the COCO dataset [35]. Unless otherwise specified, YOLO26x-seg and YOLO11x-seg were used as the front-end instance segmenters to provide high-quality initial detections and masks.

For the input resolution settings, considering that the KITTI MOTS scenario has a wide field of view and many small targets, the inference long side of the front-end segmenter is set to 1280 pixels. For MOTS20, the original video resolution is used for inference under normal conditions; for extremely dense sequences (e.g., MOTS20-07), the inference resolution is increased to 1280 pixels to mitigate the loss of small target information caused by downsampling and improve pedestrian recall rates.

### 4.2. Datasets and Evaluation Metrics

To comprehensively verify the effectiveness of TrackRefine, we evaluate it on two representative MOTS benchmark datasets, MOTS20 [36] and KITTI MOTS [37].

MOTS20 focuses on high-density pedestrian scenes and includes 4 training sequences and 4 test sequences. This dataset features significant crowd occlusion, dense target distribution, and frequent non-rigid deformations, which impose high demands on the algorithm’s robustness to occlusion, trajectory continuity, and mask segmentation accuracy.

KITTI MOTS is designed for real-world autonomous driving scenarios, consisting of 21 training sequences and 29 test sequences, with categories including Car and Pedestrian. Compared with MOTS20, KITTI MOTS features more complex scene structures, camera self-motion, and lighting changes, providing a better evaluation of the algorithm’s spatiotemporal perception and cross-scenario generalization ability.

We use the following metrics to quantitatively evaluate the algorithm’s performance: sMOTSA, MOTSA, MOTSP, IDF1, IDSW, Frag, and HOTA [5]. Among them, sMOTSA jointly measures detection, segmentation, and tracking performance; MOTSA mainly measures detection and temporal association accuracy; MOTSP reflects the average mask overlap quality of successfully matched targets; IDF1 evaluates identity consistency at the trajectory level; IDSW and Frag measure identity switches and trajectory fragmentation, respectively; and HOTA provides an overall balance between detection and association from a comprehensive perspective.

It is worth noting that in the MOTSChallenge, sMOTSA has been used as the primary ranking metric, while the KITTI MOTS official page currently uses HOTA as the main ranking metric. Therefore, in the analysis of the KITTI dataset, the Frag metric is replaced with HOTA in accordance with the current official evaluation protocol. For fairness and comparability in quantitative evaluations, all experimental and the evaluation results reported in this paper are taken from the original papers, public benchmarks, and official pages [37,38].

### 4.3. Comparison Experiments on the MOTS20 and KITTI MOTS Dataset

#### 4.3.1. MOTS20 Dataset

The quantitative comparison results of TrackRefine and existing MOTS methods on the MOTS20 test set are shown in Table 1.

As shown in Table 1, TrackRefine achieves strong performance on the MOTS20 benchmark. In particular, the TrackRefine (YOLO26x) configuration achieves the best sMOTSA of 69.4 and the lowest Frag value of 478 among all compared methods, indicating that the proposed framework effectively improves segmentation-tracking consistency and reduces trajectory fragmentation. Meanwhile, TrackRefine (YOLO11x) achieves the highest MOTSA of 83.3 and the best IDF1 of 65.8 within the proposed configurations, demonstrating favorable identity preservation and stable trajectory association performance.

Compared with representative methods such as OPITrack, TrackRefine (YOLO26x) improves sMOTSA from 63.5 to 69.4 and reduces Frag from 769 to 478, showing great improvements in trajectory continuity under crowded pedestrian scenarios. Similarly, compared with ZXPointTrack, the proposed framework achieves better segmentation-tracking consistency while maintaining competitive identity association performance.

Compared with earlier methods such as TrackRCNN and CCPNet, TrackRefine achieves significantly higher sMOTSA and MOTSA scores, indicating that the proposed lightweight mask refinement, multimodal memory modeling, and progressive association strategy effectively improve both mask quality and long-term trajectory stability in challenging MOTS scenarios with heavy occlusion and dense target interaction.

#### 4.3.2. KITTI MOTS Dataset

To further verify the applicability of TrackRefine in autonomous driving scenarios, we evaluate it on the KITTI MOTS dataset for both the Car and Pedestrian categories, as shown in Table 2.

As seen from Table 2, TrackRefine achieves competitive performance in both categories. For the Pedestrian category, TrackRefine (YOLO26x) achieves an sMOTSA of 62.4, outperforming several representative methods such as OPITrack and PointTrack. Meanwhile, the corresponding MOTSA reaches 78.0, indicating favorable trajectory continuity and segmentation-tracking consistency in challenging pedestrian scenes with frequent occlusion, appearance variation, and non-rigid motion.

For the Car category, TrackRefine achieves a MOTSP of 88.0, which is higher than those of TrackRCNN, OPITrack, and PointTrack. This result suggests that the proposed lightweight mask refinement module effectively improves boundary fitting accuracy for rigid objects in autonomous-driving scenarios. In addition, TrackRefine obtains reasonable HOTA scores on both categories, outperforming some earlier baselines while still showing a gap compared with association-optimized methods such as Seg2Track-SAM2 and OPITrack under complex driving environments involving camera motion, scale variation, and dense object interaction.

Despite these competitive results, TrackRefine still exhibits some gaps compared to the top-performing end-to-end or heavily optimized methods. For instance, in the Car category, the sMOTSA of TrackRefine (YOLO26x) is slightly lower than that of OPITrack (73.7 vs. 78.0), indicating that highly integrated joint optimization strategies can still yield marginally higher overall MOTS performance. Similarly, OPITrack achieves higher HOTA values in the Pedestrian category, reflecting a potential for improvement in handling extremely crowded and occluded scenes.

Compared with methods relying on heavy end-to-end optimization or dataset-specific retraining, TrackRefine maintains a plug-and-play and decoupled architecture that can be integrated with different front-end instance segmenters without additional joint training. Despite this design, the proposed framework still achieves competitive quantitative performance across multiple metrics, indicating that the introduced progressive association strategy and multimodal memory mechanism effectively improve tracking stability and long-term identity consistency in practical MOTS scenarios.

#### 4.3.3. Statistical Reliability Analysis on MOTS20

To further evaluate the statistical reliability of the proposed method, we conduct a sequence-level performance dispersion analysis on the MOTS20 test set. The performance of TrackRefine on each individual test sequence is reported in Table 3. The arithmetic mean, standard deviation, and coefficient of variation (CV), which is computed as the ratio of the standard deviation to the arithmetic mean across sequences, are further calculated across the four test sequences to assess whether the overall performance is consistently maintained under different crowd densities, occlusion conditions, and motion patterns.

As shown in Table 3, TrackRefine achieves stable performance across the four MOTS20 test sequences. Specifically, the sequence-level results reach 69.3 ± 2.8 in sMOTSA, 83.3 ± 2.8 in MOTSA, and 65.0 ± 7.9 in IDF1. The corresponding CV values are 4.1%, 3.4%, and 12.2%, respectively. The relatively low CV values of sMOTSA and MOTSA indicate that the proposed framework maintains stable segmentation-tracking accuracy across different sequences, rather than relying on performance gains from a single favorable sequence. Although IDF1 shows slightly larger variation, the overall identity consistency remains within a reasonable range considering the substantial differences in target density and occlusion severity among MOTS20 sequences.

Compared with percentage-based metrics, IDSW and Frag are absolute count-based indicators and therefore are not included in the CV analysis. From the sequence-level results, the absolute counts of IDSW and Frag exhibit larger inter-sequence variations. This is expected because these two metrics are strongly affected by sequence-specific factors, such as sequence length, crowd density, target interaction frequency, and the severity of long-term occlusion. Overall, the sequence-level statistical analysis supports the reliability and robustness of TrackRefine on the MOTS20 benchmark.

It should also be noted that the official combined results are computed by aggregating all sequence-level tracking results over the entire test set, rather than by simply averaging the metrics of individual sequences. Therefore, the official combined scores may differ slightly from the arithmetic mean reported in the statistical analysis.

#### 4.3.4. Qualitative Visualization Results

**Figure 5:**
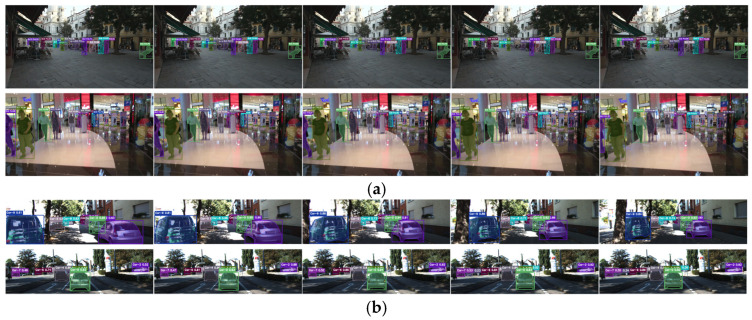
Qualitative visualization results of TrackRefine. Different colors denote different tracking IDs, with the same color representing the same tracked object across consecutive frames. (**a**) MOTS20. (**b**) KITTI MOTS.

To provide a more intuitive understanding of the tracking and segmentation performance, qualitative visualization results for the MOTS20 and KITTI MOTS datasets are shown in Figure 5.

Figure 5a presents the visualization results on the MOTS20 dataset. In crowded pedestrian scenarios with dense targets, partial occlusion, and complex background interference, TrackRefine generates temporally consistent instance-level masks and identity annotations while maintaining stable trajectory continuity for most visible targets. These observations qualitatively support the improvements in sMOTSA, MOTSA, and Frag reported in Table 1.

Figure 5b shows the visualization results on the KITTI MOTS dataset. Compared with MOTS20, KITTI MOTS contains autonomous-driving scenarios with camera motion, scale variation, illumination changes, and small distant objects. TrackRefine can still produce reliable mask-level tracking results for both cars and pedestrians. The results indicate that the proposed mask refinement and progressive association strategy help preserve object boundaries and trajectory consistency under complex road scenes, which is consistent with the quantitative results reported in Table 2.

**Figure 6:**
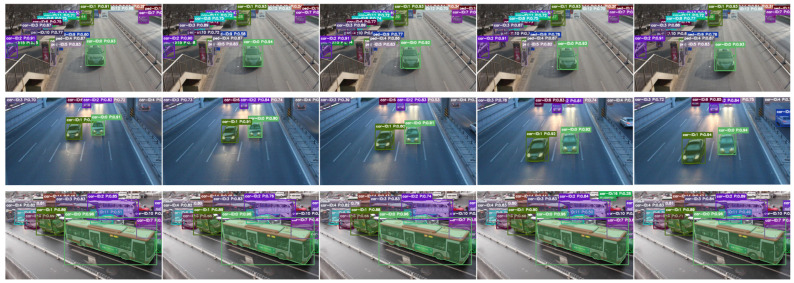
Qualitative visualization results for the UA-DETRAC dataset under daytime, nighttime, and rainy-weather conditions. Different colors denote different tracking IDs, with the same color representing the same tracked object across consecutive frames.

**Figure 7:**
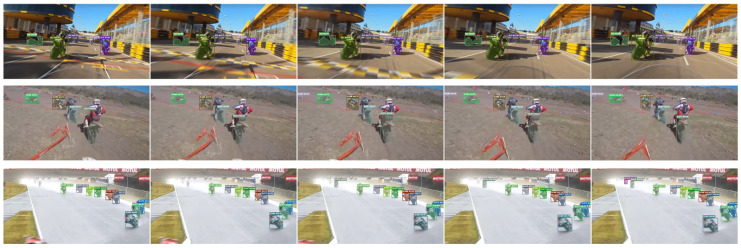
Visualization results of TrackRefine for different motorcycle racing sequences under normal, sandy-track, and rainy-weather conditions. Different colors denote different tracking IDs, with the same color representing the same tracked object across consecutive frames.

To further verify the practical applicability of TrackRefine under non-ideal conditions, additional visualization results are provided for the UA-DETRAC dataset and three motorcycle racing videos, as shown in Figure 6 and Figure 7, respectively.

As shown in Figure 6, the UA-DETRAC visualization results include daytime, nighttime, and rainy-weather traffic scenes with illumination variation, low visibility, and complex vehicle interactions. Under these challenging conditions, TrackRefine can still maintain stable trajectory continuity and identity association for most visible targets, demonstrating its applicability to realistic traffic surveillance and autonomous-driving scenarios. Nevertheless, in extremely crowded scenes with heavy occlusion, as shown in the third row of Figure 6, occasional identity switches and trajectory fragmentation may still occur.

In addition to the UA-DETRAC results, we further evaluate TrackRefine on motorcycle racing sequences. These sequences include normal racing conditions, sandy-track scenarios, and rainy-weather conditions, where motorcycles undergo high-speed motion, close-range interactions, appearance variations, and frequent occlusions. As shown in Figure 7, TrackRefine effectively tracks motorcycle targets across frames, maintaining trajectory continuity and reliable identity association in all three representative racing scenarios. These visualizations further demonstrate TrackRefine’s reliability in real-world scenarios and its plug-and-play capability across different tracking objectives. It should be noted that the UA-DETRAC and motorcycle racing sequences do not provide official pixel-level segmentation ground-truth annotations required for MOTS evaluation. Therefore, standard MOTS quantitative metrics cannot be computed, and these experiments are mainly used to qualitatively validate the plug-and-play capability of TrackRefine across different scenarios and tracking objectives.

### 4.4. Performance Analysis with Different Front-End Instance Segmenters

To provide a more transparent discussion of fairness with baseline methods, we evaluate TrackRefine under multiple front-end configurations instead of reporting only the strongest segmenter. Specifically, we keep all back-end modules unchanged, including mask refinement, memory update, and the progressive three-stage association strategy, and replace only the front-end instance segmenter. The evaluated front-ends include three Ultralytics-based models, namely YOLOv8x-seg, YOLO11x-seg, and YOLO26x-seg, as well as three additional architectures, i.e., YOLOv9c-seg, YOLOv9e-seg, and RF-DETR 2XL. The results are summarized in Table 4.

As shown in Table 4, the performance of TrackRefine varies across different front-end instance segmenters, indicating that the quality of the initial detections and masks remains an important factor in the final MOTS results. Among the Ultralytics-based models, sMOTSA increases from 65.8 with YOLOv8x-seg to 67.3 with YOLO11x-seg and further to 69.4 with YOLO26x-seg, showing that TrackRefine can benefit from stronger front-end perception. However, the identity-related metrics do not follow the same monotonic trend: YOLO11x-seg achieves the highest IDF1 of 65.8, whereas YOLO26x-seg obtains the best sMOTSA but a slightly lower IDF1 of 63.9. This suggests that stronger front-end segmenters mainly improve detection and mask quality, while identity consistency still depends on the robustness of the back-end association and trajectory recovery modules.

Therefore, TrackRefine is evaluated with both YOLO11x-seg and YOLO26x-seg in the comparison with existing methods. YOLO11x-seg provides a more balanced setting for comparison, while YOLO26x-seg represents the best-performing configuration of the complete pipeline. Reporting both configurations helps distinguish the contribution of the proposed back-end enhancement framework from the performance gain introduced by a stronger front-end segmenter.

Table 4 also provides a more practical analysis of the plug-and-play property of TrackRefine across different architectures. When replacing the front-end with YOLOv9c-seg, YOLOv9e-seg, and RF-DETR 2XL, the back-end pipeline can still run directly without any redesign or parameter adjustment. Among them, YOLOv9e-seg achieves competitive performance, with 66.4 sMOTSA, 66.6 IDF1, and 81.9 MOTSA, while YOLOv9c-seg shows slightly lower but still stable results. In contrast, the performance with RF-DETR 2XL drops more noticeably, especially in MOTSA and IDSW. Nevertheless, TrackRefine still maintains a complete tracking-segmentation process under this architecture, which demonstrates that the proposed framework is not tied to a specific segmenter family.

### 4.5. Component Ablation Study

#### 4.5.1. Results Analysis

To further analyze the contribution of each key component in TrackRefine, we conduct ablation studies on the MOTS20 test set. The ablation experiments focus on three aspects: mask refinement, memory representation, and association strategy. In particular, we progressively add the proposed modules to a basic configuration using only appearance features, and then compare the effects of lightweight mask refinement, semantic and shape cues in the memory bank, and the proposed progressive three-stage association strategy. Unless otherwise specified, all ablation experiments are conducted using YOLO26x-seg as the front-end instance segmenter. The results are reported in Table 5.

As shown in Table 5, the proposed progressive three-stage association strategy plays an important role in improving the overall tracking-segmentation performance. Under the basic appearance-only setting without mask refinement, replacing the two-stage association with the three-stage association improves sMOTSA from 62.2 to 65.8 and MOTSA from 75.0 to 79.7. Meanwhile, IDSW decreases from 626 to 512, and Frag is substantially reduced from 763 to 513. This indicates that the progressive association strategy is more effective than the conventional two-stage matching scheme in handling missed detections, short-term interruptions, and trajectory recovery in crowded scenes.

The effectiveness of mask refinement can be observed from two complementary comparisons. First, under the appearance-only and three-stage association setting, adding mask refinement improves sMOTSA from 65.8 to 68.0, IDF1 from 57.4 to 62.4, and MOTSA from 79.7 to 80.9, while reducing IDSW from 512 to 359. This shows that refined masks not only improve segmentation quality but also provide cleaner foreground regions for feature extraction and association. Second, when semantic and shape cues are both enabled, removing mask refinement decreases sMOTSA from 69.4 to 68.4 and increases Frag from 478 to 491. Although IDSW is slightly lower without mask refinement, the complete model achieves better sMOTSA, IDF1, MOTSA, and trajectory continuity, confirming the complementary role of mask refinement in the full framework.

The contribution of multimodal memory representation is also evident. Starting from the configuration with mask refinement, appearance features, and three-stage association, introducing semantic features improves sMOTSA from 68.0 to 69.1, IDF1 from 62.4 to 63.4, and MOTSA from 80.9 to 82.4. This suggests that semantic information provides additional discriminative cues beyond appearance features, which is beneficial in dense pedestrian scenarios with similar visual appearances. Further incorporating shape features increases sMOTSA to 69.4, IDF1 to 63.9, and MOTSA to 82.7. Meanwhile, IDSW decreases from 362 to 325 and Frag decreases from 511 to 478 after adding shape cues, indicating that mask-derived shape information contributes to identity preservation and trajectory continuity.

When all feature components and mask refinement are enabled, the three-stage association further improves sMOTSA from 65.1 to 69.4 and MOTSA from 77.7 to 82.7 compared with the two-stage association setting. IDSW is also reduced from 401 to 325, demonstrating that the proposed association strategy can effectively suppress identity switches. Although the two-stage setting obtains a slightly lower Frag value, its much lower sMOTSA and MOTSA indicate weaker overall detection–segmentation association consistency.

#### 4.5.2. Discussion

In online MOTS, trajectories usually evolve through three typical association states: reliable short-term continuation, temporary mismatch or weakly matched transition, and long-term lost-track recovery. A conventional two-stage association strategy mainly distinguishes high-confidence matching from supplementary matching. However, such a design tends to merge transition buffering and long-term recovery into the same supplementary association stage, making it difficult to distinguish short-term ambiguous trajectories from long-term lost trajectories. In contrast, the proposed three-stage strategy provides a more explicit decomposition of the association process. The first stage focuses on high-confidence geometric matching for active trajectories, the second stage serves as a transition buffer with candidate filtering, and the third stage performs long-term recovery under stricter spatial and memory-based constraints. This design separates short-term continuation, intermediate uncertainty, and long-term reactivation into three functionally different matching phases, thereby reducing priority conflicts among trajectories with different states. This analysis also supports the comparison results in Section 4.3, where TrackRefine achieves competitive overall performance on both MOTS20 and KITTI MOTS, further confirming that the observed improvements stem from the collaborative effect of the proposed modules rather than any single component.

We also considered whether a finer four-stage association scheme would be necessary. From a design perspective, an additional association stage would mainly further subdivide the transition or recovery process without introducing new discriminative cues. Such a scheme would increase the number of matching rounds and hyperparameters, while also increasing the risk of over-fragmenting candidate sets during online inference. Therefore, the three-stage strategy can be regarded as a balanced design: it is more expressive than the conventional two-stage scheme, while avoiding the additional complexity of a finer multi-stage cascade. The ablation results in Table 5 support this opinion, as the three-stage setting achieves the best overall sMOTSA and MOTSA with fewer identity switches.

Overall, the ablation results demonstrate that each component contributes to the final performance from different perspectives. The three-stage association strategy mainly improves global tracking-segmentation consistency and reduces identity switches; mask refinement enhances foreground quality and stabilizes subsequent feature extraction; semantic and shape cues enrich the memory representation and improve identity discrimination. With all modules enabled, TrackRefine achieves the best overall performance, reaching 69.4 sMOTSA, 63.9 IDF1, and 82.7 MOTSA, with 325 IDSW and 478 Frag on the MOTS20 test set.

### 4.6. Hyperparameter Ablation Study

To further evaluate the robustness of TrackRefine to different hyperparameter settings, we conduct a hyperparameter ablation study on the MOTS20 dataset. The analysis focuses on five representative groups of parameters that are closely related to the proposed modules: the memory-bank-oriented trajectory update and confirmation thresholds, the dynamic IoU weights used in progressive association, the memory update momentum coefficient α, the instance-overlap suppression threshold $\theta_{m}$, and the maximum RoI size $R_{max}$ used in Fast GrabCut.

In TrackRefine, the memory bank is regulated by two trajectory-level thresholds: the trajectory update threshold and the trajectory confirmation threshold. The trajectory update threshold determines whether a candidate trajectory is eligible to update its memory representation, the trajectory confirmation threshold further determines whether the trajectory is reliable enough to be confirmed and maintained for subsequent memory-guided association. To evaluate their influence, we consider three representative threshold settings: (0.3, 0.4), (0.4, 0.5), and (0.5, 0.6), where the first value denotes the trajectory update threshold and the second value denotes the trajectory confirmation threshold. Table 6 reports the performance of TrackRefine under different memory-bank-oriented trajectory threshold settings.

As shown in Table 6, as the memory-bank-oriented trajectory thresholds gradually increase, IDSW and Frag generally decrease. For example, when the thresholds are set to (0.5, 0.6), IDSW is reduced to 291 and Frag is reduced to 428, indicating that a stricter memory-bank update control strategy helps improve identity stability and trajectory continuity. This is because only reliable trajectories are used to update and maintain memory representations, thereby reducing the risk of introducing ambiguous or noisy features into the memory bank. Nevertheless, this improvement in stability is achieved at the cost of lower object coverage, as reflected by the decrease in sMOTSA and MOTSA to 66.7 and 79.1, respectively. Some valid but low-confidence candidate trajectories may fail to update their memory features or be confirmed in time, which reduces the coverage of short-term, occluded, or weak-response targets.

In summary, the memory-bank-oriented trajectory update and confirmation thresholds control the trade-off between memory coverage and memory reliability. Lower thresholds allow more candidate trajectories to participate in memory-bank feature accumulation, thereby improving object coverage and trajectory recall, but they may also introduce less reliable features into the memory bank. In contrast, higher thresholds maintain cleaner and more reliable trajectory prototypes, but may suppress valid short-term or partially occluded trajectories from contributing to memory updating. Based on the sMOTSA result, we adopt (0.3, 0.4) as the default configuration of TrackRefine.

We further analyze the influence of the IoU-based spatial constraint in the joint association module of the second stage. In this stage, the matching score between the i-th trajectory and the j-th detection is computed by combining spatial overlap, appearance similarity, shape similarity, and short-term memory similarity:

(11) $${score}_{ij}=w_{iou}\cdot {IoU}_{ij}+w_{app}\cdot {Sim}_{ij}^{app}+w_{sha}\cdot {Sim}_{ij}^{sha}+w_{stm}{\cdot Sim}_{ij}^{stm},$$

where ${IoU}_{ij}$ denotes the spatial overlap similarity between the trajectory and the detection. ${Sim}_{ij}^{app}$, ${Sim}_{ij}^{sha}$, and ${Sim}_{ij}^{stm}$ denote the appearance similarity, shape similarity, and short-term memory similarity, respectively. The four weights satisfy $w_{iou}+w_{app}+w_{sha}+w_{stm}=1.$

To adapt the association criterion to different mismatch states, we define the mismatch duration Δt as the number of frames elapsed since the trajectory was last successfully matched. Under the default setting, the dynamic weight allocation is defined as:

(12) $$(w_{iou},w_{app},w_{sha},w_{stm})=\left\{\begin{array}{cc} (0.45,\;0.30,\;0.10,\;0.15), & 0\leq \Delta t\leq 1,\\ (0.30,\;0.30,\;0.25,\;0.15), & 1<\Delta t\leq 5,\\ (0.15,\;0.35,\;0.35,\;0.15), & 5<\Delta t\leq 20. \end{array}\right.,$$

In the second stage, for recently unmatched trajectories (0 ≤ Δ*t* ≤ 1), the predicted position is still relatively reliable; therefore, IoU is assigned the largest weight 0.45. This threshold prevents spatial overlap from dominating the association decision and allows the combined appearance, shape, and short-term memory cues to correct imperfect geometric predictions. As the mismatch duration increases, the reliability of spatial overlap gradually decreases due to target deformation, occlusion, and camera motion. Accordingly, the IoU weight is reduced from 0.45 to 0.30 and then to 0.15, while the contribution of shape and appearance cues is increased to support feature-based recovery.

Table 7 reports the sensitivity analysis of the IoU-based spatial constraint weights in joint association. It should be noted that $w_{1}$, $w_{2}$, and $w_{3}$ in the table denote the IoU weights used in the three mismatch stages, namely $w_{1}=w_{iou}^{0\leq \Delta t\leq 1}$, $w_{2}=w_{iou}^{1<\Delta t\leq 5}$, and $w_{3}=w_{iou}^{5<\Delta t\leq 20}$.

When the IoU schedule changes from (0.25,0.20,0.15) to (0.45,0.30,0.15), sMOTSA increases from 67.5 to 69.4, and MOTSA increases from 80.3 to 82.7. This indicates that a stronger spatial prior in the early mismatch stage helps suppress ambiguous associations and improves the consistency between detection, segmentation, and tracking. However, the corresponding IDSW and Frag values also increase from 305 and 438 to 325 and 478, respectively. This suggests that stronger spatial constraints improve overall matching accuracy but may reduce the flexibility of trajectory continuation in some occlusion or deformation cases.

In addition to the above parameters, we also evaluate the sensitivity of three module-specific hyperparameters, namely α, $\theta_{m}$, and $R_{max}$, which are associated with memory updating, instance-overlap suppression, and local mask refinement, respectively. The results are summarized in Table 8.

As shown in Table 8, when the momentum coefficient α increases from 0.1 to 0.9, sMOTSA changes from 69.3 to 69.4, while IDF1 increases from 63.5 to 63.9. This can be attributed to the pre-update filtering constraints of the long-term prototypes. Specifically, only observations from confirmed trajectories that pass the quality-aware gating, occlusion filtering, and motion-consistency checking are written into the long-term prototypes. According to Equation (8), the coefficient α determines the relative contribution of the historical long-term prototype and the current detection feature during the update process. When α = 0.9, the historical long-term prototype accounts for a larger proportion in the update process, resulting in a more stable identity representation. Therefore, α = 0.9 is adopted as the default setting in the subsequent experiments.

For the instance-overlap suppression threshold, $\theta_{m}=0.7$ achieves the best overall performance. When $\theta_{m}$ is reduced to 0.5, the suppression strategy becomes more aggressive, which helps reduce the duplicate instances and improves IDF1 while reducing IDSW. However, overly aggressive suppression may also remove highly overlapped but valid adjacent instances in crowded scenes, leading to increased trajectory fragmentation and a decrease in sMOTSA and MOTSA. In contrast, increasing $\theta_{m}$ to 0.9 makes the suppression strategy more conservative. Although this setting preserves more candidate masks, redundant or highly overlapped masks are more likely to remain, which may introduce additional ambiguity during data association.

For the maximum RoI size $R_{max}$, increasing the value from 128 to 256 improves sMOTSA from 68.7 to 69.4 and IDF1 from 63.1 to 63.9. This indicates that an excessively small RoI may discard useful local context required by Fast GrabCut, thereby limiting boundary refinement quality and subsequent association reliability. When $R_{max}$ is further increased to 384, sMOTSA and MOTSA remain unchanged, and IDF1 only slightly increases from 63.9 to 64.0, whereas Frag increases from 478 to 485. This suggests that enlarging the refinement region does not necessarily bring additional segmentation-tracking gains and may introduce more background pixels into the local optimization. In addition, the computational cost also increases noticeably with a larger RoI. The mean latency of the Fast GrabCut module increases from 105.9 ms at $R_{max}$ = 256 to 170.5 ms at $R_{max}$ = 384. Considering both tracking performance and computational efficiency, $R_{max}$ = 256 is adopted as the default setting in all experiments.

### 4.7. Computational Complexity and Latency Analysis

To further evaluate the deployment efficiency of TrackRefine and quantify the additional overhead introduced by the proposed back-end modules, we conduct a module-wise computational complexity and latency analysis. The analysis focuses on the three main back-end components of TrackRefine: Fast GrabCut refinement, the long-short-term memory bank, and the progressive three-stage association strategy.

For the Fast GrabCut refinement module, the complexity is defined as

(13) $$C_{\mathrm{gc}}=O\left(\sum_{i}I_{gc}R_{i}\right),$$

where $I_{gc}$ denotes the number of GrabCut iterations and $R_{i}$ denotes the number of pixels in the local RoI of the i-th instance. Since the proposed refinement strategy performs graph-cut optimization within bounded local RoIs rather than over the whole frame, its computational cost is mainly determined by the number of detected instances and their cropped RoI sizes. This design avoids the high cost of full-frame iterative GrabCut while preserving local boundary refinement capability.

For the memory bank, the complexity is formulated as

(14) $$C_{mem}=O(QLd),$$

where Q is the number of memory queries, L is the memory size, and d is the feature dimension.

For the progressive three-stage association strategy, the complexity is defined as

(15) $$C_{match}=O(NMd+NMR_{mask}+n^{3}),$$

where N and M denote the numbers of trajectories and detections, respectively, $R_{mask}$ denotes the mask-level comparison cost, and n=max(N,M) corresponds to the scale of Hungarian assignment.

The measured latency of each module is reported in Table 9. All measurements are obtained on the MOTS20-02 sequence over 600 frames after excluding warm-up frames. The minimum, maximum, and mean latency values are reported to reflect both the average runtime efficiency and the variation under different scene complexities.

As shown in Table 9, the progressive three-stage association module is the dominant source of back-end latency, with a mean latency of 285.4 ms and a maximum latency of 583.6 ms. This result is consistent with its theoretical complexity, since the module involves trajectory-detection feature comparison, mask-level similarity computation, candidate filtering, and Hungarian assignment. In addition, the relatively large latency variation indicates that the association overhead changes with the number of targets, active trajectories, and occlusion interactions across frames. In particular, frames with denser targets and more potential matching candidates usually introduce higher computational costs during association.

The Fast GrabCut refinement module introduces a moderate but bounded additional cost. Its mean latency is 105.9 ms, while the maximum latency reaches 172.5 ms. Since the proposed refinement strategy performs graph-cut optimization only within local RoIs rather than over the whole frame, the computational overhead remains effectively constrained. This suggests that the local refinement design improves mask boundary quality without introducing excessively expensive full-frame optimization.

The memory bank exhibits the lowest latency among the three back-end modules, with a mean latency of 9.6 ms and a maximum latency of 33.5 ms. Although its theoretical complexity scales with the number of memory queries, memory size, and feature dimension, the actual runtime overhead remains relatively small in practice. However, the overall mean latency of the full system reaches 400.9 ms, indicating that the real-time performance still requires further improvement.

**Figure 8:**
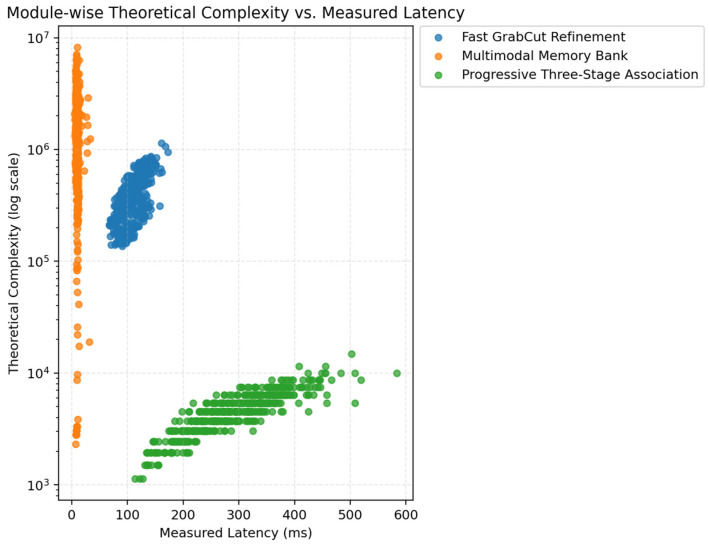
Theoretical complexity versus measured latency of the three back-end modules.

Each point corresponds to a module call after warm-up frames. The y-axis is displayed on a logarithmic scale because the theoretical complexity values of the different modules span different numerical ranges. Figure 8 further visualizes the relationship between theoretical complexity and measured latency for the three back-end modules. The results show that theoretical complexity and practical runtime exhibit consistent overall trends but are not strictly proportional. In particular, the association module contributes most significantly to practical latency because it combines multiple matching stages and assignment operations, whereas the Fast GrabCut refinement and memory bank modules remain within relatively stable latency ranges.

## 5. Conclusions and Future Work

In this work, TrackRefine is presented as a plug-and-play decoupled enhancement framework for online MOTS, aiming to strengthen tracking-by-segmentation pipelines while preserving their modular design. TrackRefine enhances overall system performance through three key modules: lightweight mask refinement, quality-aware multimodal memory modeling, and a progressive three-stage association strategy. Experimental results on MOTS20 show that TrackRefine achieves 69.4 sMOTSA, 82.7 MOTSA, and 478 Frag, demonstrating robust performance in challenging scenes. On KITTI MOTS, it achieves 62.4/73.7 sMOTSA and 78.0/85.4 MOTSA for Pedestrian and Car, respectively, indicating good robustness and cross-scenario generalization capability. In addition, experiments with different front-end configurations further demonstrate that TrackRefine can stably adapt to various instance segmenters. Ablation studies show that the progressive three-stage association strategy and the multimodal memory bank are the main contributors to the improvements in overall segmentation-tracking performance, while the mask refinement module mainly improves instance boundary quality and related segmentation metrics.

Despite these promising results, several limitations should be noted more explicitly. First, although TrackRefine achieves particularly strong fragmentation performance, its IDF1 remains inferior to some offline MOTS methods. Second, HOTA performance remains less competitive, suggesting room for improvement in balancing detection, localization, and association quality. Third, the real-time performance of TrackRefine still requires further improvement. Future research may consider confidence calibration, trajectory-level post-processing, and uncertainty-aware prediction to reduce false positives, missed detections, and association ambiguity. In addition, incorporating more robust temporal consistency constraints and cross-frame quality estimation may help improve the alignment between detection and association performance.

## Figures and Tables

**Table 1:** Quantitative Comparison Results on the MOTS20 Test Set. ↑ indicates that higher values represent better performance, whereas ↓ indicates that lower values represent better performance. Bold numbers indicate the best value for each metric, while underlined numbers indicate the second-best value.

Method	sMOTSA (↑)	IDF1 (↑)	MOTSA (↑)	IDSW (↓)	Frag (↓)
TrackRCNN [5]	40.6	42.4	55.2	576	868
MPNTrackSeg [16]	58.7	**68.8**	73.7	**202**	858
CCPNet [39]	59.3	58.1	75.5	484	645
PointTrack [17]	62.3	42.9	76.8	541	868
OPITrack [18]	63.5	45.4	75.5	342	769
TrackRefine (YOLO11x)	67.3	65.8	**83.3**	324	508
TrackRefine (YOLO26x)	**69.** **4**	63.9	82.7	325	**4** **78**

**Table 2:** Quantitative Comparison Results on the KITTI MOTS Test Set. ↑ indicates that higher values represent better performance, whereas ↓ indicates that lower values represent better performance. Bold numbers indicate the best value for each metric, while underlined numbers indicate the second-best value.

Method	sMOTSA (↑)		MOTSP (↑)		MOTSA (↑)		IDSW (↓)		HOTA (↑)	
	Car	Ped	Car	Ped	Car	Ped	Car	Ped	Car	Ped
TrackRCNN [5]	67.0	47.3	85.1	74.6	79.7	66.1	692	482	56.6	41.9
STC-Seg [40]	66.2	42.6	82.8	75.6	81.1	57.7	676	408	62.8	43.9
Seg2Track-SAM2 [41]	68.8	49.7	86.2	77.4	81.0	68.1	**95**	**79**	**74.1**	60.0
OPITrack [18]	78.0	61.1	87.2	**81.3**	90.4	75.8	542	234	73.0	**60.4**
PointTrack [17]	**78.5**	61.5	87.1	81.0	**90.9**	76.5	346	176	62.0	54.4
TrackRefine (YOLO11x)	74.1	59.8	87.0	78.8	85.5	76.7	366	374	66.7	51.4
TrackRefine (YOLO26x)	73.7	**62.4**	**88.0**	80.7	85.4	**78.0**	494	363	66.9	53.5

**Table 3:** Sequence-level performance and statistical reliability analysis on the MOTS20 test set. ↑ indicates that higher values represent better performance, whereas ↓ indicates that lower values represent better performance.

Sequence	sMOTSA (↑)	IDF1 (↑)	MOTSA (↑)	IDSW (↓)	Frag (↓)
MOTS20-01	67.8	62.4	85.5	23	33
MOTS20-06	73.1	64.0	85.7	164	203
MOTS20-07	66.6	57.5	80.1	96	176
MOTS20-12	69.8	76.2	81.7	44	69
Mean ± Std.	69.3 ± 2.8	65.0 ± 7.9	83.3 ± 2.8	81.8 ± 62.8	120.3 ± 82.0
CV (%)	4.1	12.2	3.4	-	-
Official Combined	69.4	63.9	82.7	325	478

**Table 4:** Performance analysis with different front-end instance segmenters on the MOTS20 test set. ↑ indicates that higher values represent better performance, whereas ↓ indicates that lower values represent better performance. Bold numbers indicate the best value for each metric.

Segmenter	sMOTSA (↑)	IDF1 (↑)	MOTSA (↑)	IDSW (↓)	Frag (↓)
RF-DETR 2XL	58.2	62.0	74.2	543	571
YOLOv8x-seg	65.8	65.5	81.6	312	490
YOLOv9c-seg	64.5	64.6	80.1	337	574
YOLOv9e-seg	66.4	**66.6**	81.9	**302**	**466**
YOLO11x-seg	67.3	65.8	**83.3**	324	508
YOLO26x-seg	**69.** **4**	63.9	82.7	325	478

**Table 5:** Ablation Experiment Results for the MOTS20 Test Set. ↑ indicates that higher values represent better performance, whereas ↓ indicates that lower values represent better performance. Bold numbers indicate the best value for each metric, while underlined numbers indicate the second-best value.

Mask Refinement	Memory Bank			Association		sMOTSA (↑)	IDF1 (↑)	MOTSA (↑)	IDSW (↓)	Frag (↓)
	Appearance	Semantic	Shape	Two-Stage	Three-Stage					
✗	✓	✗	✗	✓	✗	62.2	56.5	75.0	626	763
✗	✓	✗	✗	✗	✓	65.8	57.4	79.7	512	513
✓	✓	✗	✗	✗	✓	68.0	62.4	80.9	359	509
✓	✓	✓	✗	✗	✓	69.1	63.4	82.4	362	511
✓	✓	✓	✓	✗	✓	**69.** **4**	**63.** **9**	**82.7**	32 5	4 78
✗	✓	✓	✓	✗	✓	68.4	63.6	82.6	**322**	491
✓	✓	✓	✓	✓	✗	65.1	**63.9**	77.7	401	**472**

**Table 6:** Effect of memory-bank-oriented trajectory update and confirmation thresholds on the MOTS20 dataset. ↑ indicates that higher values represent better performance, whereas ↓ indicates that lower values represent better performance.

Memory Bank Update Threshold Setting	sMOTSA (↑)	IDF1 (↑)	MOTSA (↑)	IDSW (↓)	Frag (↓)
(0.3, 0.4)	69.4	63.9	82.7	325	478
(0.4, 0.5)	68.5	64.6	81.5	312	460
(0.5, 0.6)	66.7	64.2	79.1	291	428

**Table 7:** Sensitivity analysis of spatial constraint weights in joint association on the MOTS20 test set. ↑ indicates that higher values represent better performance, whereas ↓ indicates that lower values represent better performance.

Dynamic IoU Weights ($w_{1},w_{2},w_{3}$)	sMOTSA (↑)	IDF1 (↑)	MOTSA (↑)	IDSW (↓)	Frag (↓)
(0.25, 0.20, 0.15)	67.5	63.1	80.3	305	438
(0.30, 0.20, 0.10)	68.5	63.9	81.5	315	464
(0.45, 0.30, 0.15)	69.4	63.9	82.7	325	478

**Table 8:** Sensitivity analysis of α, $\theta_{m}$, and $R_{max}$ on MOTS20. ↑ indicates that higher values represent better performance, whereas ↓ indicates that lower values represent better performance.

Parameter	Value	sMOTSA (↑)	IDF1 (↑)	MOTSA (↑)	IDSW (↓)	Frag (↓)
α	0.1	69.3	63.5	82.7	327	478
α	0.5	69.3	63.9	82.6	330	485
α	0.9 (default)	69.4	63.9	82.7	325	478
$\theta_{m}$	0.5	69.3	64.5	82.6	322	487
$\theta_{m}$	0.7 (default)	69.4	63.9	82.7	325	478
$\theta_{m}$	0.9	69.0	63.9	82.4	362	477
$R_{max}$	128	68.7	63.1	82.5	331	483
$R_{max}$	256 (default)	69.4	63.9	82.7	325	478
$R_{max}$	384	69.4	64.0	82.7	327	485

**Table 9:** Module-wise computational complexity and latency analysis.

Module	Min Latency (ms)	Max Latency (ms)	Mean Latency (ms)
Fast GrabCut Refinement	67.4	172.5	105.9
Memory Bank	5.01	33.5	9.6
Three-Stage Association	113.6	583.6	285.4

## Data Availability

The data used in this study are publicly available from the corresponding public datasets cited in the manuscript. The source code is available at https://github.com/RobotIt/TrackRefine (accessed on 8 June 2026). Further information is available from the corresponding author upon reasonable request.

## Supplementary Materials

The following supporting information can be downloaded at https://www.mdpi.com/article/10.3390/s26123696/s1, Table S1: Feature representation and memory module parameters; Table S2: Mask refinement and trajectory management parameters; Table S3: Progressive three-stage association parameters; Algorithm S1: Lightweight Mask Refinement; Algorithm S2: Quality-Aware Long–Short-Term Memory Update; Algorithm S3: High-Confidence Geometric Matching; Algorithm S4: Transition-Buffer Matching; Algorithm S5: Long-Term Lost-Track Recovery.

## Abbreviations

The following abbreviations are used in this manuscript:

MOTS	Multi-Object Tracking and Segmentation
MOT	Multi-Object Tracking
IoU	Intersection over Union
HOTA	Higher Order Tracking Accuracy
MOTSA	Multi-Object Tracking and Segmentation Accuracy
sMOTSA	soft Multi-Object Tracking and Segmentation Accuracy
IDSW	Identity Switches
Frag	Fragmentations
