# Supplementary material for: TrackRefine: A Plug-and-Play Decoupled Enhancement Framework for Online Multi-Object Tracking and Segmentation
Source: Sensors (Basel). 2026 Jun 10;26(12):3696. doi: 10.3390/s26123696 (PMC13306713; doi:10.3390/s26123696)
Supplement: Supplementary file 1 [file sensors-26-03696-s001.zip › sensors-4330429-supplementary.pdf]

Supplementary Materials

Supplementary Materials for "TrackRefine: A Plug-and-Play Decoupled Enhancement Framework for Online Multi-Object Tracking and Segmentation".

These Supplementary Materials provide the key parameter configurations, reproduction steps, and detailed algorithmic procedures used in the experiments. Unless otherwise specified, all back-end parameters were kept fixed across datasets and front-end replacement experiments. For front-end replacement experiments, only the instance segmenter weights were changed, while the proposed mask refinement, memory, and association modules remained unchanged.

S.1. Key Parameter Configurations

Tables S1-S3 summarize the parameters used for multimodal representation and memory modeling, mask refinement and trajectory management, and progressive association. The default front-end listed in Table S1 corresponds to the main reported configuration; alternative front-end segmenters can be used by replacing the weights in the command line.

Table S1. Feature representation and memory module parameters.

Module	Parameter	Value	Description
Front-end instance segmenter	Default weights	YOLO26x-seg	Used as the default front-end instance segmenter
Appearance feature extractor	Model	OSNet-x1.0	Used to extract ReID appearance features
Appearance feature	Dimension	512	Appearance embedding dimension
Semantic feature	Dimension	512	RoI-level semantic representation
Shape feature	Dimension	256	Mask-shape representation extracted by the mask encoder
Joint semantic-shape feature	Dimension	256	Output dimension after multimodal projection and encoding
Memory encoder	Input dimension	768	Concatenation of semantic and shape features
Memory encoder	Output dimension	256	Compact joint representation
Short-term memory	Window length	7 frames	Stores recent temporal observations
Long-term memory	Maximum capacity	50 frames	Stores long-term identity information
Long-term prototype	Maximum prototypes per track	5	Retains high-quality prototype features
Prototype update	EMA momentum	0.9	Smoothly updates long-term prototypes
Quality-aware gating	Detection quality threshold	0.3	Filters unreliable observations before memory writing
Motion gating	Velocity threshold	50.0	Suppresses abnormal motion observations

Table S2. Mask refinement and trajectory management parameters.

Module	Parameter	Value	Description
Mask suppression	Mask IoU threshold	0.7	Removes highly overlapping duplicate masks
Fast GrabCut refinement	Maximum local RoI resolution	256 × 256	Controls local graph-cut computation cost
Fast GrabCut refinement	Iteration number	1	Performs single-iteration local refinement
Fast GrabCut refinement	RoI padding	20 pixels	Adds local context around each instance
Morphological trimap	Kernel size	Adaptive, minimum 3	Used for erosion and dilation in trimap generation
Occlusion detection	Box IoU threshold	0.3	Detects overlapping trajectories
Occlusion detection	Area history length	5 frames	Determines mask-area variation under occlusion
Detection filtering	Detection threshold	0.15	Minimum score for candidate detections
New trajectory initialization	New-track threshold	0.30	Minimum score for creating a new trajectory
Tentative trajectory confirmation	Confirmation threshold	0.40	Score threshold for confirming tentative tracks
Trajectory lifetime	Maximum lost frames	20	Removes inactive trajectories after long absence
Tentative trajectory lifetime	Maximum tentative age	2 frames	Removes unstable tentative trajectories
Track interpolation	Maximum interpolation gap	15 frames	Fills short trajectory gaps
Track stitching	Maximum time gap	40 frames	Allows post-hoc trajectory stitching
Track stitching	Distance threshold	150 pixels	Spatial constraint for trajectory stitching

Table S3. Progressive three-stage association parameters.

Stage	Parameter	Value	Description
Stage 1	Box IoU weight	0.3	Weight for bounding-box IoU
Stage 1	Mask IoU weight	0.7	Weight for mask-level IoU
Stage 1	Matching threshold	0.5	Minimum IoU for high-confidence matching
Stage 2, 0≤lost age ≤ 1	Weights: IoU, ReID, shape, STM	0.45, 0.30, 0.10, 0.15	Geometry-dominant matching for short-term unmatched tracks
Stage 2, 1 < lost age ≤ 5	Weights: IoU, ReID, shape, STM	0.30, 0.30, 0.25, 0.15	Balanced matching between geometry and appearance-shape cues
Stage 2, 5 < lost age ≤ 20	Weights: IoU, ReID, shape, STM	0.15, 0.35, 0.35, 0.15	Appearance- and shape-dominant matching for longer mismatches
Stage 2	IoU gate	0.15	Candidate retained if IoU is sufficiently high
Stage 2	ReID gate	0.35	Candidate retained if ReID similarity is sufficiently high

Stage 2	Shape gate	0.45	Candidate retained if shape similarity is sufficiently high
Stage 3	Initial recovery similarity threshold	0.35	Minimum score for lost-track recovery
Stage 3, lost age ≤ 5	Minimum IoU gate	0.03	Spatial gate for short-term lost tracks
Stage 3, lost age > 5	Minimum IoU gate	0.01	Relaxed spatial gate for long-term lost tracks
Stage 3, lost age ≤ 5	ReID weight	0.75	Combines ReID and deep memory similarity
Stage 3, 5 < lost age ≤ 15	ReID weight	0.90	Increases reliance on long-term appearance memory
Stage 3, lost age > 15	ReID weight	0.97	Strongly relies on ReID prototype for long-term recovery

S2. Detailed Algorithmic Procedures

Algorithms S1-S5 summarize the execution logic of the three core modules: lightweight mask refinement, quality-aware long-short-term memory update, and progressive three-stage association from high-confidence geometric matching to long-term lost-track recovery.

S2.1 Lightweight Mask Refinement

The complete procedure of the proposed Lightweight Mask Refinement is summarized in Algorithm S1. Given the current frame, the initial bounding box, and the initial mask, the method first restricts the optimization to a padded local RoI. A trimap is then automatically generated from the initial mask through morphological erosion and dilation, where reliable foreground, reliable background, and uncertain boundary regions are explicitly separated. A single graph-cut optimization is finally performed within the local RoI, and the refined mask is mapped back to the original image coordinate system.

Algorithm S1. Lightweight Mask Refinement

Input: Image $\boldsymbol{I_t}$, bounding box $\boldsymbol{b_i^t}$, initial mask $\boldsymbol{m_i^t}$, maximum RoI size $\boldsymbol{R_{max}}$

Output: Refined mask $\hat{\boldsymbol{m}}_i^t$

Crop a local RoI $\boldsymbol{I_{roi}}$ from $\boldsymbol{I_t}$ according to $\boldsymbol{b_i^t}$ with context padding
Crop the corresponding mask region $\boldsymbol{m_{roi}}$ from $\hat{\boldsymbol{m}}_i^t$
if the size of $\boldsymbol{I_{roi}}$ exceeds $\boldsymbol{R_{max}}$ **then**
    Resize $\boldsymbol{I_{roi}}$ and $\boldsymbol{m_{roi}}$ proportionally
end if
Construct a trimap from $\boldsymbol{m_{roi}}$ by morphological operations
Set the eroded mask region as sure foreground
Set the complement of the dilated mask region as sure background
Set the remaining boundary region as unknown area
Initialize GrabCut labels using the trimap
Perform one graph-cut optimization within the local RoI
Obtain the optimized local mask $\hat{\boldsymbol{m}}_{roi}$
if the RoI was resized **then**
    Resize $\hat{\boldsymbol{m}}_{roi}$ back to the original RoI size
end if
Paste $\hat{\boldsymbol{m}}_{roi}$ back to the original image coordinate system
return $\hat{\boldsymbol{m}}_i^t$

S2.2 Enhanced Multimodal Long-Short-Term Memory Bank

Based on the multimodal memory representation and the selective write strategy, the quality-aware memory update process is summarized in Algorithm S2. For each matched trajectory, the observation quality is evaluated using the detection confidence, refined mask reliability, and motion consistency. The update strategy is then determined according to both the trajectory state and the observation quality.

Algorithm S2. Enhanced Multimodal Long-Short-Term Memory Bank

---

Input: Matched trajectory $\boldsymbol{k}$, current features $\{\boldsymbol{f}_k^{app,t}, \boldsymbol{f}_k^{joi,t}, \boldsymbol{f}_k^{sha,t}\}$, detection score $\boldsymbol{s}_k^t$, refined mask $\widehat{\boldsymbol{m}}_k^t$, trajectory state $\boldsymbol{q}_k^t$, short-term memory queue $\boldsymbol{S}_k$, long-term prototypes $\boldsymbol{\mathcal{P}}_k = \{\boldsymbol{P}_k^{app}, \boldsymbol{P}_k^{joi}\}$

Output: Updated memory state $\boldsymbol{M}_k = \{\boldsymbol{S}_k, \boldsymbol{\mathcal{P}}_k\}$

Compute observation quality:

$$\boldsymbol{Q}_k^t = \boldsymbol{Quality}(\boldsymbol{s}_k^t, |\widehat{\boldsymbol{m}}_k^t|, \boldsymbol{\Delta v}_k^t)$$

if $\boldsymbol{q}_k^t$ is tentative **then**
    Do not update long-term prototypes
    Optionally update only temporary short-term state
    return $\boldsymbol{M}_k$
end if
if $\boldsymbol{Q}_k^t < \boldsymbol{\theta}_q$ **then**
    Reject the observation for long-term memory
    Push observation into short-term memory only
    return $\boldsymbol{M}_k$
end if
Push $\boldsymbol{f}_k^{app,t}, \boldsymbol{f}_k^{joi,t}, \boldsymbol{f}_k^{sha,t}, \widehat{\boldsymbol{m}}_k^t, \boldsymbol{Q}_k^t$ into $\boldsymbol{S}_k$
if $|\boldsymbol{S}_k| > \boldsymbol{L}_s$ **then**
    Remove the oldest element from $\boldsymbol{S}_k$
end if
if $\boldsymbol{q}_k^t$ is confirmed and not severely occluded **then**
    Update long-term appearance prototype:

$$\boldsymbol{P}_k^{app,t} \leftarrow \boldsymbol{\alpha} \boldsymbol{P}_k^{app,t-1} + (\boldsymbol{1} - \boldsymbol{\alpha}) \boldsymbol{f}_k^{app,t}$$

    Update long-term joint semantic-shape prototype:

$$\boldsymbol{P}_k^{joi,t} \leftarrow \boldsymbol{\alpha} \boldsymbol{P}_k^{joi,t-1} + (\boldsymbol{1} - \boldsymbol{\alpha}) \boldsymbol{f}_k^{joi,t}$$

else if $\boldsymbol{q}_k^t$ is occluded **then**
    if $\boldsymbol{t}$ satisfies low-frequency update interval **then**
        Conservatively update long-term prototypes using same momentum rule
    end if
end if
Retain only top-$\boldsymbol{K}_p$ high-quality prototype samples if prototype pool is full
return $\boldsymbol{M}_k$

S2.3 Progressive Three-Stage Association Strategy

S2.3.1 High-Confidence Geometric Matching

The first-stage high-confidence geometric matching is summarized in Algorithm S3. This stage only considers active trajectories with strong temporal continuity and uses normalized bounding-box IoU and mask IoU to construct the cost matrix. After Hungarian assignment, an additional absolute bounding-box IoU constraint is applied to avoid unreliable matches caused by relative score normalization.

Algorithm S3. Stage 1: High-Confidence Geometric Matching

Input: Current detections $\widehat{\mathcal{D}}_t$, active trajectories $\mathcal{T}_a$
Output: Matched pairs $\mathcal{A}_t^{(1)}$ and unmatched detections $\mathcal{U}_t^{(1)}$
Initialize $\mathcal{A}_t^{(1)} \leftarrow \emptyset$
Initialize $\mathcal{U}_t^{(1)} \leftarrow \widehat{\mathcal{D}}_t$
for each active trajectory $\boldsymbol{\tau_i} \in \boldsymbol{T_a}$ and detection $\boldsymbol{d_j} \in \boldsymbol{U_t^{(1)}}$ **do**
    Compute bounding-box IoU: $\mathbf{IoU}_{box}(\boldsymbol{i},\boldsymbol{j})$
    Compute mask IoU: $\mathbf{IoU}_{mask}(\boldsymbol{i},\boldsymbol{j})$
end for
Normalize similarity matrices:

$$\widetilde{\text{IoU}}_{box} = \boldsymbol{Norm}(\mathbf{IoU}_{box})$$
$$\widetilde{\text{IoU}}_{mask} = \boldsymbol{Norm}(\mathbf{IoU}_{mask})$$

Construct first-stage cost matrix:

$$\boldsymbol{C_{ij}^{(1)}} = \mathbf{1} - \boldsymbol{\lambda_1}\widetilde{\text{IoU}}_{\mathbf{box}} - \boldsymbol{\lambda_2}\widetilde{\text{IoU}}_{mask},$$

Obtain assignments using Hungarian algorithm
for each assigned pair $(\boldsymbol{\tau_i}, \boldsymbol{d_j})$ **do**
    if $\boldsymbol{IoU_{box}(i,j)} \geqslant \boldsymbol{\theta_{box}^{(1)}}$ **then**
        Add $(\boldsymbol{\tau_i}, \boldsymbol{d_j})$ to $\mathcal{A}_t^{(1)}$
        Remove $\boldsymbol{d_j}$ from $\mathcal{U}_t^{(1)}$
    end if
end for
return $\mathcal{A}_t^{(1)}, \mathcal{U}_t^{(1)}$

S2.3.2 Transition-Buffer Matching

The second-stage transition-buffer matching is summarized in Algorithm S4. Unlike conventional supplementary matching that simply relaxes thresholds, this stage acts as both a matching transition and a candidate filtering stage. According to the mismatch age of each trajectory, the matching weights are dynamically adjusted from geometry-dominated to appearance- and shape-dominated configurations. Candidate pairs that violate basic feasibility constraints are filtered out before assignment.

Algorithm S4. Stage 2: Transition-Buffer Matching

Input: Unmatched trajectories $\mathcal{T}_r$, unmatched detections $\mathcal{U}_t^{(1)}$, memory bank $\mathcal{M}$
Output: Matched pairs $\mathcal{A}_t^{(2)}$and unmatched detections $\mathcal{U}_t^{(2)}$
Initialize $\mathcal{A}_t^{(2)} \leftarrow \emptyset$
Initialize $\mathcal{U}_t^{(2)} \leftarrow \mathcal{U}_t^{(1)}$
for each trajectory $\boldsymbol{\tau_i} \in \mathcal{T}_r$ and detection $\boldsymbol{d_j} \in \mathcal{U}_t^{(2)}$ **do**
    Query long-term prototypes and short-term memory from M
    Compute geometric cost $\boldsymbol{C_{ij}^{iou}}$
    Compute appearance cost $\boldsymbol{C_{ij}^{app}}$ using the ReID prototype
    Compute shape cost $\boldsymbol{C_{ij}^{sha}}$ using mask-shape similarity
    Compute short-term memory cost $\boldsymbol{C_{ij}^{stm}}$ using memory fusion
    Compute the mismatch age:

$$\boldsymbol{\Delta t_i} = \boldsymbol{t} - \boldsymbol{t_i^{last}}$$

    if $\boldsymbol{\Delta t_i} \leq \mathbf{1}$ **then**
        Use geometry-dominated weights
    else if $\mathbf{1} < \boldsymbol{\Delta t_i} \leq \mathbf{5}$ **then**
        Increase the weight of shape information

else
    Use appearance- and shape-dominated weights
end if
Construct the second-stage cost:
$$\boldsymbol{C}_{ij}^{(2)} = \boldsymbol{w}_{iou}\boldsymbol{C}_{ij}^{iou} + \boldsymbol{w}_{app}\boldsymbol{C}_{ij}^{app} + \boldsymbol{w}_{sha}\boldsymbol{C}_{ij}^{sha} + \boldsymbol{w}_{stm}\boldsymbol{C}_{ij}^{stm}$$
if the pair fails the basic feasibility constraint **then**
    Set $\boldsymbol{C}_{ij}^{(2)} \leftarrow \mathbf{1}$
end if
end for
Obtain assignments using the Hungarian algorithm
for each assigned pair ($\boldsymbol{\tau}_i$, $\boldsymbol{d}_j$) **do**
if $\boldsymbol{C}_{ij}^{(2)} < \boldsymbol{\theta}_c^{(2)}$ **then**
    Add ($\boldsymbol{\tau}_i$, $\boldsymbol{d}_j$) to $\boldsymbol{\mathcal{A}}_t^{(2)}$
    Remove $\boldsymbol{d}_j$ from $\boldsymbol{\mathcal{U}}_t^{(2)}$
end if
end for
return $\boldsymbol{\mathcal{A}}_t^{(2)}$, $\boldsymbol{\mathcal{U}}_t^{(2)}$

S2.3.3 Long-Term Lost-Track Recovery

The long-term lost-track recovery process is summarized in Algorithm S5. For long-term unmatched trajectories, the method first applies strict spatial reachability constraints, including bounding-box IoU and center-distance thresholds, before evaluating memory-based similarity. Only detections satisfying these spatial constraints are compared with long-term appearance prototypes and historical memory, which reduces the risk of incorrect trajectory reactivation after long occlusion.

Algorithm S5. Stage 3: Long-Term Lost-Track Recovery

Input: Lost trajectories $\mathcal{T}_l$, unmatched detections $\mathcal{U}_t^{(2)}$, memory bank $\mathcal{M}$
Output: Recovered matched pairs $\mathcal{A}_t^{(3)}$ *and* final unmatched detections $\mathcal{U}_t$
Initialize $\mathcal{A}_t^{(3)} \leftarrow \emptyset$
Initialize $\mathcal{U}_t \leftarrow \mathcal{U}_t^{(2)}$
for each lost trajectory $\tau_i \in \mathcal{T}_l$ **do**
Query long-term appearance prototype and historical memory from $\mathcal{M}$
Initialize best similarity $S^* \leftarrow \theta_{rec}$
Initialize best detection $d^* \leftarrow \emptyset$
for each unmatched detection $d_j \in \mathcal{U}_t$ **do**
    Compute bounding-box IoU: $\mathrm{IoU}_{box}(i,j)$
    Compute center-point distance:
$$\mathrm{Dist}_{center}(i,j) = \| \mathrm{p}_i^{pred} - \mathrm{p}_j^{det} \|_2$$
    if $\mathrm{IoU}_{box}(i,j) < \theta_{iou}^{rec}$ or $\mathrm{Dist}_{center}(i,j) > \theta_{dist}^{rec}$ **then**
        Continue
    end if
    Compute prototype appearance similarity S_{ij}^{app}
    Compute memory-fusion similarity S_{ij}^{mem}
    Compute recovery similarity:
$$S_{ij}^{rec} = \beta_i S_{ij}^{app} + (1 - \beta_i) S_{ij}^{mem}$$

if $S_{ij}^{rec} > S^*$ **then**
$S^* \leftarrow S_{ij}^{rec}$
$d^* \leftarrow d_j$
end if
end for
if $d^* \neq \emptyset$ **then**
Reactivate τ_i using d^*
Add $(\tau_i,\ d^*)$ to $\mathcal{A}_t^{(3)}$
Remove d^* from $\mathcal{U}_t$
end if
end for
return $\mathcal{A}_t^{(3)}, \mathcal{U}_t$

S.3. Reproducibility Notes

To improve reproducibility, all experiments should use the same model weights, dataset split, input resolution, and parameter settings listed in Tables S1–S3. For runtime comparison, the GPU model, CUDA version, PyTorch version, and batch/inference settings should be reported. Since TrackRefine is an online framework, frames are processed sequentially without using future-frame information. The front-end instance segmenter is used as a replaceable module, and the proposed refinement, memory, and association modules are applied only in the back-end tracking stage.
